# Mental health issues in unaccompanied refugee minors

**DOI:** 10.1186/1753-2000-3-13

**Published:** 2009-04-02

**Authors:** Julia Huemer, Niranjan S Karnik, Sabine Voelkl-Kernstock, Elisabeth Granditsch, Kanita Dervic, Max H Friedrich, Hans Steiner

**Affiliations:** 1 Medical University of Vienna, Department of Child and Adolescent Psychiatry, Waehringer Guertel 18-20, A-1090 Vienna, Austria; 2 University of California San Francisco School of Medicine, Department of Anthropology, History & Social Medicine, 3333 California Street, Suite 485, San Francisco, CA 94143, USA; 3 Stanford University School of Medicine, Child Psychiatry and Child Development, Department of Psychiatry and Behavioral Sciences, 401 Quarry Road, Stanford, CA, 94305-5719, USA

## Abstract

Previous studies about unaccompanied refugee minors (URMs) showed that they are a highly vulnerable group who have greater psychiatric morbidity than the general population. This review focuses on mental health issues among URMs. Articles in databases PsycINFO, Medline and PubMed from 1998 to 2008 addressing this topic were reviewed. The literature had a considerable emphasis on the assessment of PTSD symptoms. Results revealed higher levels of PTSD symptoms in comparison to the norm populations and accompanied refugee minors. In several studies, age and female gender predicted or influenced PTSD symptoms. The existing literature only permits limited conclusions on this very hard to reach population. Future research should include the analysis of long-term outcomes, stress management and a more thorough analysis of the whole range of psychopathology. Additionally, the development of culturally sensitive norms and standardized measures for diverse ethnic groups is of great importance.

## Introduction

By the end of 2007, the total population under the responsibility of the United Nations High Commissioner for Refugees (UNHCR) amounted to 31.7 million. Among this large population, nearly 44% are under 18 years of age and 10% are under the age of 5 [1]. Unaccompanied refugee minors are a hidden population within many of these statistics and are increasingly regarded as an important group. In order to synthesize the limited number of publications and bring some uniformity to this area of research and intervention, the current review initially provides definitions of different refugee and asylum seeking populations, and subsequently presents a summary of the available research on mental health issues among unaccompanied refugee children and adolescents. Finally, results are critically analyzed and directives for future research and interventions are provided.

### Definitions

UNHCR's founding mandate defines refugees as people who are outside their country and cannot return owing to a well-founded fear of persecution because of their race, religion, nationality, political opinion or membership of a particular social group. When people flee from their own country and seek safety in another state, they often have to apply for 'asylum' – which is the right to be recognized as bona fide refugee and receive legal protection and material assistance [2].

The term 'refugee' refers to all people who are subsumed under the 1951 Convention relating to the Status of Refugees, its 1967 Protocol, and the 1969 OAU Convention Governing the Specific Aspects of Refugee Problems in Africa. This group relates to those people who fulfill the UNHCR Statute as well as individuals with "complementary forms of protection" and "temporary protection." Complimentary protection signifies a formal permission under national law, established on humanitarian grounds to people who need international protection to reside in a country, although they might not fulfill refugee status under the conventional refugee criteria. Temporary protection is provided for those people who arrive in large numbers due to conflicts and violence and who subsequently don't require formal or individual status determination [1].

"Asylum-seekers are individuals who have sought international protection and whose claim for refugee status has not yet been determined [1]". When looking at underage asylum seekers, internationally used definitions of "child" and "unaccompanied/separated children" have been central to establish their status; these definitions may differ from culturally variable approaches towards the term 'child'. The word 'child' is used throughout the guidelines on the "Formal Determination of the Best Interests of the Child" [3] in accordance with the definition determined in Article 1 of the Convention of the Right of the Child (CRC) [4]. The CRC states that "a child means every human being below the age of 18 years unless, under the law applicable to the child, majority is attained earlier" [4]. "Unaccompanied children and adolescents (or unaccompanied minors) – according to the CRC – are children and adolescents who have been separated from both parents and relatives and are not being cared for by an adult who, by law or custom, is responsible for doing so [4]".

"Separated children and adolescents are those separated from both parents, or from their previous legal or customary primary care-giver, but not necessarily from their relatives. These may therefore include young children accompanied by adult family members other than their parents [4]". All member states of the UN, except the United States of America and Somalia, have ratified the CRC [4]. In the US, the enactment of the Refugee Act of 1980 and the Immigration and Nationality Act [5] define "unaccompanied refugee minor" (URM) in a similar way to the CRC, as a child who is under the age of 18, and "who entered the United States unaccompanied by and not destined to (a) a parent or (b) a close non-parental adult relative who is willing and able to care for the child or (c) an adult with a clear and court verifiable claim to custody of the minor; and who has no parent(s) in the United States" [5].

### Statistical overview

Additional file 1 summarizes the number of separated children and adolescents seeking asylum in Europe in 2006 based on data provided by the "Separated Children in Europe Programme" (SCEP) [6].

In the US, 7000 to 9000 unaccompanied children and adolescents (referring primarily to those, who have entered the United States illegally and who are not being considered as refugees) have been referred to the Office of Refugee Resettlement (ORR) by the Department of Homeland Security since 2005 [7]. In total, 782 new URMs were enrolled in a programme specifically for unaccompanied refugee youth (Unaccompanied Refugee Minor Program) between 1999 and 2005 [see additional file 2] [8]. During this time period, Sub-Saharan Africa, Central America and the Caribbean, and the Middle East represented the originating regions for the majority of URMs in the US. The programme was initially developed in the 1980s to address the needs of thousands of children originating from Southeast Asia without a guardian to care for them. Since its inception, almost 12,000 minors have entered the programme [8].

### Intervention patterns

In the United States URMs are treated according to the principles of the Unaccompanied Refugee Minor Program, which is part of the United States Refugee Program [9]. The United States Refugee Program provides resettlement and foster care services for URMs. When URMs are admitted to the United States, the ORR is responsible to provide care for them. This is guaranteed until they reach the age of majority or until they are reunited with their families. ORR works with state and local service providers, as well as volunteer agencies, to establish foster placement, services, etc. ORR and the state and volunteer services cooperate with foster homes, group homes, independent living, semi-independent living, possible relatives, and residential treatment facilities to find placement for URMs [9].

In Europe, SCEP was established in 1997 as a reaction to the situation of separated children and adolescents in the continent. The network aims at improving the situation of this vulnerable group by means of research, policy analysis and advocacy at national and regional levels. SCEP was founded by UNHCR and the International Save the Children Alliance based on the complementary mandates and areas of expertise of the two organizations. Its membership includes 25 European Union (EU) countries as well as Bulgaria, Croatia, Norway and Switzerland. In terms of URMs and separated children and adolescents, UNHCR also cooperates with other networks, such as Le Réseau Euro-Méditerranéen pour la Protection des Mineurs Isolés (REMI) and the Council of the Baltic Sea States which have organized expert groups on children and adolescents. SCEP also publishes a newsletter on regular basis containing statistical data as well as political, legislative and intervention information.

### Mental health issues among URMs

The vulnerability of the group makes the relatively small number of URMs arriving in Europe and the Unites States notable. Several reviews have examined mental health issues among refugee minors [10-12]. The number of studies assessing psychopathology parameters among URMs is comparably small. To the knowledge of the authors, this is the first review which exclusively examines the mental health status of URMs.

URMs have diverse demographic origins; they leave their home countries for a number of reasons. They lack a familial system at a crucial developmental period and have experienced multiple stressful events due to problems in their home countries, their refuge process and the situation in the country of asylum.

Most of the research to date has examined URMs from the perspective of trauma and post-traumatic stress. This review aims to critically evaluate this literature, examine gaps in the existing research base, and proposes a set of possible approaches for the future of this subfield.

## Methods

We reviewed 22 papers from 1998 to 2008 [see additional file 3] addressing mental health issues among URMs. Literature was gathered from databases including PsycINFO, Medline and PubMed. We examined papers that clearly defined this population as the primary object of study and also used empirical techniques to provide overviews and study this population. Qualitative and quantitative papers were included.

## Results

When looking at recent research on URMs, five types of study designs were found. In this section, we review the existing research by grouping the studies based on their design and approach.

### 1. Comparing URMs to accompanied minors or non-refugees

Hodes, Jagdev et al. [13] compared 78 unaccompanied refugee youth, predominantly from the Horn of Africa and the Balkans, between 13 and 18 years of age, with a group of 35 accompanied refugee youth, living in London, UK. By means of the Birleson Depression Self-Rating Scale, the Impact of Event Scale (IES) and the Harvard Trauma Questionnaire, they found that unaccompanied refugee adolescents had been affected by greater war trauma and losses and had elevated posttraumatic stress symptoms. The mean number of trauma events was 28 (SD 10.4) among the unaccompanied refugee youth and 12 (SD 9.2) among the accompanied refugee adolescents. Male URMs had a mean IES score of 37 (SD 13.0), whereas male accompanied refugee minors scored 15 (SD 20.6). The IES score among females was 42 (SD 14.5) and 22 (SD 16.6), respectively. Thirty-two of 52 (61.5%) male URMs and only 2 of 14 male accompanied refugee minors had a high risk of developing posttraumatic stress disorder (p = .005). Significantly more female URMs (19 of 26 (73.1%)) than female accompanied refugee minors (6 of 17 (35.3%)) had an elevated risk for posttraumatic stress disorder (p = .032). Low-support living circumstances, number of traumatic experiences, increasing age, and gender predicted posttraumatic symptoms among unaccompanied refugee youth. The authors concluded that high-support living arrangements and perspectives after the age of 18 years were needed to ameliorate psychological distress.

Bean et al. [14] compared psychological distress, traumatic stress reactions, and experiences of URMs with adolescents accompanied by parents. The sample of the study included 920 URMs (48 countries; 12–18 years), 1059 immigrant and refugee adolescents (111 countries; 13–18 years), and 1294 Dutch adolescents (10 secondary and 3 trade schools throughout the Netherlands). The Hopkins Symptom Checklist-37 for Adolescents (HSCL-37A), Stressful Life Events (SLE), and Reactions of Adolescents to Traumatic Stress (RATS) questionnaires were translated into the most prevalent languages of URMs in the Netherlands. The authors found that gender moderated internalizing emotional problems and externalizing behaviour in the two comparison groups but not among URMs. In addition, the older the adolescent, the more emotional problems and the more experienced stressful life events were reported. URMs showed a higher number of stressful life events in comparison to the other groups. The scores of SLE, RATS and the Hopkins Symptom Checklist were 6.1 (SD = 2.7), 49.1 (SD = 11.6) and 65.7 (SD = 14.4) among URMs, whereas 3.3 (SD = 2.5), 37.6 (SD = 10.8) and 56.2 (SD = 12.7) among accompanied immigrant and refugee adolescents and 3.0 (SD = 2.1), 31.8 (SD = 8.7) and 57.8 (SD = 10.7) among Dutch adolescents. Across all samples, the event "loss of loved one" was most frequently reported. URMs reported exceptionally (statistically significant) high levels of exposure to physical and sexual maltreatment compared with the other groups. The authors also found that stressful life events were the strongest predictor of internalizing behaviour and traumatic stress reactions.

Wiese and Burhorst [15] compared URMs with those refugee minors accompanied by families, referred to a child and adolescent psychiatry service in the Netherlands in 2003 and 2004. Their sample involved 129 patients of which 70 had families and 59 were unaccompanied. For this study, data was taken from the intake report, and was included in a structured questionnaire, which involved the following: (1) demographic data: age, sex, family (family and social conditions – age, education and occupation of parents), religion, ethnicity, living conditions (in the home-country and in the Netherlands), education (in the home-country and currently), work, social information (friends, relatives, etc.), duration of stay in the country, reason for asylum, how he/she travelled to the country, and asylum status; (2) registration of the main traumatic experiences; (3) psychological symptoms as described by the client, parents, guardian and teacher; (4) diagnostic hypothesis as specified by the treatment coordinator, according to DSM-IV. Sexual abuse was more frequent among unaccompanied minors (36%) compared to the group of children with families (7%). Sixty-seven percent of the unaccompanied refugee girls and 14% of the boys had experienced sexual abuse.

Extreme traumatic events, such as having witnessed the killing of parents, living on the streets, or being kidnapped and living with rebels, were experienced by 6% of the children with families and 25% of unaccompanied children (*p *= .002). In terms of the number of traumatic experiences, 54% mentioned one to three traumatic events, and 37% reported four or more traumatic experiences. Unaccompanied refugee minors (63%) were more likely to have been victim to four or more traumatic events than children and adolescents with families (16%). URMs showed a significantly higher prevalence of depressive disorder (47% vs. 27%, *p *< .001), borderline personality disorder (22 vs. 9%; *p *= .045), and psychosis (15 vs. 1%; *p *= .005) when being compared to minors with families.

Derluyn et al. [16] compared migrant (N = 1249) and native Belgian (N = 602) adolescents (ages 11 to 18 yrs). The following methods were used: Hopkins Symptom Checklist-37 for Adolescents (HSCL-37A), Stressful Life Events (SLE), Strengths and Difficulties Questionnaire (SDQ) and Reactions of Adolescents to Traumatic Stress (RATS). Migrant adolescents experienced an average of 3.64 (SD = 2.74) traumatic events. This is significantly more (t = 8.493; df = 1549; p < 0.001, 95% CI = 0.75–1.19) than non-migrant adolescents (mean = 2.67; SD = 2.02). Non-migrants and migrants differed significantly from each other on several subscales [see additional file 4]. Girls displayed more anxiety symptoms (HSCL-37A) (p < 0.02), more emotional problems (SDQ) (p < 0.02) and higher avoidance scores (RATS) (p < 0.03), while boys had more problems in pro-social behaviour (SDQ) (p < 0.001). The living situation affected the following domains significantly: anxiety symptoms (HSCL-37A), conduct problems (SDQ), and pro-social behaviour (SDQ). The number of traumatic experiences (SLE) significantly predicted all subscales (hyperactivity and pro-social behaviour: p < 0.002, peer problems: p < 0.004, other subscales: p < 0.001).

Additionally, gender significantly influenced the prevalence of emotional and behavioural problems, with girls being more vulnerable. Generally, little overall differences concerning the prevalence of emotional and behavioural problems between migrant and Belgian adolescents were found. URMs reported more emotional problems and more symptoms of anxiety, depression and PTSD, but less conduct problems than accompanied migrant adolescents. According to the results of the study, the duration of stay in Belgium did not influence the prevalence of emotional and behavioural problems among migrant adolescents.

To address the need for mental health care and the patterns of utilization of mental health care services, information from unaccompanied refugee minors (n = 920), their legal guardians (n = 557), and their teachers (n = 496) was collected [17]. The following instruments were used: RATS, HSCL-37A, SLE, Mental Health Questionnaire for guardians, Mental Health Questionnaire for teachers, Child Behavior Checklist (CBCL), Teacher Report Form (TRF). The well-being, need and utilization of mental health care services of URMs was compared to those of a representative Dutch adolescent sample (n = 1059). URMs who reported a mental health care need also displayed higher levels of emotional distress when being compared to Dutch adolescents who reported a similar need for mental health care. Guardians and teachers were able to detect emotional distress and mental health care needs in only a small proportion (30%) of URMs. The referral of URMs to mental health care services seems to be strongly influenced by the need and emotional distress as observed by guardians. Consequently, 48.7% of the total sample of URMs reported that their need for mental health care was not sufficiently met.

Loughry et al. [18] examined the behavioural and emotional problems of former unaccompanied refugee children and adolescents who had repatriated to Vietnam from refugee centres in Hong Kong and South East Asia. Data was collected using the Achenbach Youth Self-Report (YSR), the Cowen Perceived Self-Efficacy scale, a Social Support scale as well as an Exposure to Trauma scale. URMs were compared with a matched sample of non-refugee children and adolescents who had never left Vietnam. The study population included 455 Vietnamese children and adolescents aged between 10 and 22 years; 238 of them had formerly resided in refugee camps without their parents. Results revealed that YSR results of unaccompanied and non-unaccompanied children differed [*F *(3,451) = 5.50, *p *= .001] on the externalizing score but not on the internalizing or total problem scores. The mean YSR internalizing score among URMs was 5.89 (SD = 4.34) and 7.09 (SD = 5.20) among the non-refugee group. Those who described their current standard of living as 'very difficult' reported the highest internalizing scores [*F *(3,448) = 7.32, *p *< .001], and those adolescents with higher socioeconomic means showed the highest externalizing scores [*F *(3,448) = 8.43, *p *< .001].

Perceived self-efficacy, number of social supports, and experience of social support did not differ between the two groups of youths. Further analysis revealed that a significant interaction between the immigration status of the children and adolescents and their subjective perception of their current standard of living explained the differences in the YSR. As discussed by the authors, the results suggest that the experience of living without parents in a refugee camp does not lead to increased behavioural and emotional problems in the immediate years after repatriation.

Sourander [19] examined the traumatic events and behaviour symptoms of 46 unaccompanied refugee minors who waited for placement in an asylum center in Finland. All the clinical information available about the refugee children's experiences before and during their flight as well as after their arrival in Finland was collected. Additionally, the Child Behavior Checklist (CBCL) was used. URMs in the sample had gone through a number of losses and separations, through persecution and threats. Approximately half of the minors had results within clinical or borderline range when evaluated with the CBCL. Younger age (< 15 years) was associated with more severe psychiatric problems. Sourander stated that there were not enough rehabilitative services and too little staff, and the time spent in the asylum center waiting for the placement decision was relatively long. Thus, he concluded that a high level of social support and mental health care inherent in Finland's social welfare system does not guarantee an appropriate level of care for unaccompanied refugee children.

### 2. Quantitative studies

Derluyn and Broekaert [20] reported that between 37–47% of 166 URMs suffered from severe or very severe symptoms of anxiety, depression and posttraumatic stress. Girls and those who had experienced greater numbers of traumatic events were at higher risk for the development of emotional problems. Social workers reported a high prevalence of internalizing problems and considerable externalizing problems in the study population.

Geltman et al. [21] examined the functional and behavioural health of URMs from the Sudan who were resettled in the United States. Methods included open interviews, the Child Health Questionnaire, the Harvard Trauma Questionnaire, the Ways of Coping Instrument and YSR. The study included 304 male Sudanese refugee minors enrolled in the U.S. URMs program. Results revealed that 20% of the adolescents suffered from PTSD, and these youth had significantly worse functional scores than those who had not been diagnosed with PTSD. In addition, social isolation and personal injury were associated with PTSD.

Reijneveld et al. [22] examined the effects of different reception policies on mental health of unaccompanied adolescent asylum seekers. The authors compared the behavioural and emotional problems of 69 URMs in a reception centre exclusively for minors (including a highly structured daily programme) with 53 URMs in facilities which were shared with adult asylum seekers. The first facility involved a highly structured daily programme, emphasizing restriction, the repatriation to the country of origin and limited contact to the local population. Outcomes revealed more emotional problems on the Hopkins Symptom Checklist among the youth in the restrictive reception centre (mean score of 59.3 in the restrictive setting compared to a mean score of 53.4 in the non-restrictive setting). Main effects demonstrated a rise in anxiety. Girls showed more effect than boys. Thus, a restrictive reception may affect the mental health of adolescent asylum seekers. This potential adverse effect needs to be taken into account by health care workers and policy makers according to the authors of the study.

### 3. Follow-up studies

Bean, Eurelings-Bontekoe et al. [23] examined the prevalence, course, predictors and concordance of psychological distress and behavioural problems of URMs (n = 582) in a 12 month follow-up study assessed by self-report, teacher and guardian ratings. The following measures were used: Hopkins Symptom Checklist-37 for Adolescents, Stressful Life Events (SLE), Reactions of Adolescents to Traumatic Stress (RATS), the Child Behavior Checklist Guardian report and the Teacher Report Form. The self-report questionnaires were translated into the most prevalent languages of the adolescents. The results of the study showed continuously high severity levels of psychological distress as well as behavioural problems in all 3 groups. This fact indicates a chronic course of traumatic stress reactions. Youth experienced their distress as severe (50%) and chronic in nature (stable throughout one year). This result was confirmed by reports from guardians (33%) and teachers (36%). Baseline psychopathology at the first time of assessment (n = 920) was the strongest predictor of follow-up psychopathology at the second time of assessment (n = 582) according to reports of all informants; this finding accounted for 22–51% of variance in the sample. The results did not differ from previous studies in terms of severity of traumatic stress reactions, internalizing distress and the association between trauma and high levels of psychological distress. Elevated levels of stress reactions were significantly different from the low levels reported among the normative population. The effect of socio-demographic factors on severity levels of emotional distress among minors turned out to be small or nonexistent. Age proved to be the only factor of importance. A higher number of negative life events were associated with older age. The study revealed no indication of anxiety and depression symptoms decreasing over time.

Wallin et al. [24] described how URMs experienced their own living situation and well-being seven years after they had gained permanent residency. The original sample involved 34 unaccompanied refugee minors who were placed in a municipality in Sweden. At that time, they had been between 16 and 26 years old. Eleven of these youths took part in the follow-up study after a mean of 10 years in their new country. Qualitative interviews were conducted by means of a phenomenological analysis. Most of the participants expressed satisfaction with their lives. In examining their social network, the authors found that their friends came from the same ethnic group while their Swedish contacts were mostly workmates. Some of the examined URMs felt lonely and described despair and depression. One participant reported the presence of post-traumatic stress disorder with symptoms continuing after 11 years in the new homeland. The majority of participants had worked through the problems which typically affect refugees and had started to adapt to their new country. Those who dropped out presumably live with more distress in everyday life and suffer from depression more frequently.

Spinhoven et al. [25] investigated the consistency with which stressful life events are reported by URMs during a 12-month follow-up period and analyzed to what extent demographic and psychopathology variables affected memory consistency. The questionnaires, Hopkins Symptom Checklist-37 for Adolescents, Stressful Life Events (SLE) and Reactions of Adolescents to Traumatic Stress (RATS), were used to examine 920 URMs aged 12 to 18 years old, with 63% completing the follow-up measurements. The total number of self-reported stressful events did not change between baseline (M = 4.5; SD = 1.7) and the follow-up (M = 4.5; SD = 1.9). Younger participants and those with lower levels of internalizing behaviour and posttraumatic stress at follow-up were at a higher risk of memory inconsistencies. Apart from that, younger participants and those with fewer inconsistencies were more likely to have gained a temporary residence permit.

### 4. Qualitative, intervention and health service studies

Geltman et al. [26] assessed whether mental health counseling and other health services could be correlated to functional health outcomes of unaccompanied Sudanese refugee minors in the United States. The survey comprised 304 Sudanese URMs in foster care through the U.S. URM Program. Methods included Child Health Questionnaire (CHQ) scales and questions regarding care for symptoms associated with behavioural disorders. Posttraumatic stress disorder (PTSD) was measured with the Harvard Trauma Questionnaire (HTQ). Health service questions were derived from the National Health Interview Survey. Counseling was not associated with improved health outcomes. The majority of the sample (76%) sought medical care for problems associated with behavioural and emotional issues; this was more common among those with PTSD (OR = 2.5, 95% CI = 1.004–6.26). Functional health status did not correlate with the likelihood of receiving mental health counseling. Despite this finding, youth in the study were more likely to seek care from any health professional regardless of PTSD status. Reported receipt of mental health counseling did not affect CHQ subscale scores.

Derluyn and Broekaert [27] discussed the issue of being an unaccompanied refugee child or adolescent from a legal and psychological perspective. A special emphasis was put on the situation of URMs in Belgium by presenting demographic, service and interventional perspectives. The conclusion of the article stresses the importance of integrating the psychological perspective in the elaboration process of reception and care systems for URMs. URMs should be primarily considered as children and not as refugees or foreigners as emphasized by legislative frameworks.

Bates et al. [28] examined the resettlement experiences of unaccompanied Sudanese refugee youth placed in foster care from the perspectives of the adolescents, foster parents, and agency caseworkers using qualitative techniques. The author discusses the need for increased funding to support more intensive educational services, cultural training, foster parents, school personnel and flexibility to provide services in more culturally appropriate modalities.

Goodman [29] used a case-centred, comparative, narrative approach to analyze the transcripts of 14 male URMs (ages 16–18 years) from Sudan recently resettled in the United States. The narratives were obtained during a 60 to 90 minute interview and were analyzed for form as well as for content. Four key themes concerning coping strategies were identified: (1) collectivity and the communal self – the adolescents described themselves as part of the group of refugees and stressed the importance of mutual support to endure their situation, (2) suppression and distraction – these defense mechanisms were described as a means to forget about the stressful past; suppression was also reflected in the structure of the narrative – stories about very stressful life events were told without a lot of detail and with little emotion or evaluation, (3) creating meaning – the idea that God is in power and that God's will is predominant in deciding about your life or dying was present in most of the stories, (4) emerging from hopelessness to hope – youth in the study contrasted the stressful past with the prospect of a more fortunate future in the US. The findings underline the importance of culturally sensitive approaches toward URMs and the value of examining resilience and coping in future research.

Thomas [30] analysed pre-flight experiences of unaccompanied asylum seeking children in the UK. One hundred and twenty unaccompanied separated children were chosen from the case files of a social services team providing care for 400 URMs. The information was acquired through an analysis of legal statements, case notes and interviews. Half of the study population had experienced separation from or loss of parents and/or family members (47%), and a further 41% had personally experienced or witnessed violence. Sexual violence (such as rape) was reported by 24% of African girls. Traumatic journeys to the UK were reported by many participants. The study concludes that URMs arrive in the UK with a variety of potentially traumatic experiences. According to the authors, culturally appropriate research is needed to identify health and social needs after arrival.

General considerations on providing health care for refugee children and unaccompanied minors are provided by Lynch [31]. This descriptive paper gives a broad overview of the health services in the UK, and the special challenges in addressing the needs of URMs. The author argued that especially those aged 16 to 18 years had problems in obtaining referrals to specialist providers.

Rousseau et al. [32] interviewed 10 Somali URMs (ages 13 to 18 years) as well as key informants and representatives of their host community in Canada. Topics of investigation involved the adolescents' self-report of their migratory experience, their notion of what other young men of their community experience and handle during the migratory process, and the illustration of specific stories, which the authors felt, would describe other ways of coping with the same migratory experiences. Results were presented using descriptive case reports. Concepts of resilience were discussed in the framework of two normative systems including the traditional upbringing in Somali culture and the forced exile experience in North America. The authors outlined that the individual experience of adversity needed to be mapped on to the collective meaning of stressful life events. Understanding these interfaces could broaden the definition of resilience and protection processes and might have an important influence on therapeutic approaches.

### 5. Validation of instruments

Two studies report the validation process of instruments for refugee adolescents and unaccompanied refugee minors. With a background of unmet needs in mental health care and a lack of reliable and valid instruments among immigrant/refugee adolescents, Bean et al. [33] provided preliminary psychometric properties for the HSCL-37A among four heterogenous groups – native Dutch, native Belgian and Belgian refugee/immigrant adolescents as well as Dutch URMs. Modifications were made to the instrument in order to assess internalizing and externalizing symptoms associated with reactions to trauma, and to make it accessible to culturally diverse, adolescent populations. Among the specific changes, colored circles of increasing size were added to the terms of a Likert scale. On the basis of a vocabulary list for foreign students in the Netherlands, items were adjusted to language abilities. Finally, the questionnaires were translated and presented bilingually. The confirmative two factor analyses, by language version, re-established the two factor structure of internalizing and externalizing symptoms. The total scale as well as the subscales showed good internal consistency and acceptable test-rest reliability. Cronbach's alpha for the total score was r = .9, it ranged from r = .95 to r = .84 for the language versions. Apart from that, the construct, content and criterion validity were rated as good. The authors stated that the HSCL-37A was a reliable and valid instrument among culturally diverse refugee adolescents to assess emotional distress and maladaptive behaviour.

In a second study, Bean et al. [34] examined the preliminary psychometric properties of the Reaction of Adolescents to Traumatic Stress questionnaire (RATS) among the same populations as described above. The authors carried out similar modifications as in their prior study, assessing the validity of the HSCL-37A. The confirmatory factor analyses, by language version, gave support to a three-factor structure of intrusion, avoidance/numbing, and hyperarousal. The total and subscales of the RATS showed good internal consistency and validity. Cronbach's alpha for the total score was r = .91, it ranged from r = .81 to r = .93 for the language versions. The RATS was described as a reliable and valid instrument for assessing posttraumatic stress reactions of culturally diverse adolescents.

## Discussion

URMs are a highly vulnerable group who likely suffers from more psychiatric morbidity than comparable populations. Several studies have shown that female gender and age are important factors predicting or influencing posttraumatic stress symptoms. URMs are not only a small group within society, but also a population that has been neglected in terms of research and interventions. They also constitute a rising population with specific legislative, psychosocial and psychiatric needs. Lustig et al. [10] describes the necessity of further research and intervention among this population, while also emphasizing refugee youth in detention. The saliency of these issues was also confirmed by Fazel et al. [35]. There are a relatively small number of papers which have been published in this subfield over the last decade. This may be due to the fact that URMs have limited access to health institutions as a consequence of their living circumstances and language barriers, which makes it difficult to use standardised measures to explore these adolescents in research but also to enable interventions. The very instability of their living situations and their frequent migrant movements makes organizing a research protocol difficult.

It is generally difficult to get an overview regarding migrating children, adolescents and families. Data is few and the practice of collecting data varies from country to country. When data is collected the quantitative statistical analyses can be biased by the chosen methods [36]. When approaching research and interventions in diagnostic and therapeutic procedures with URMs it is therefore important to use structured guidelines and recommendations [37].

Thomas et al. [37] underscore the following summary points: "unaccompanied youths seeking asylum are vulnerable; research is made difficult by cultural differences and practical barriers such as language; steps need to be taken to ensure that participation in research does not cause harm; consultation with young people, their carers, and service providers is vital in building trust and assessing the best way to carry out research." Service models for addressing the needs of vulnerable youth have been described in other publications [38].

It is evident that psychiatric health care for URMs needs to take into account their specific needs and problems [39]. As Lynch [31] has described, "The health of refugee children must be considered beyond ensuring access to health care to include issues such as housing and education. Refugees require support in using services, and their culture and religious background must be taken into account. Asylum seeking youths have the same rights to health as any other children and adolescents. It is important to arrange access to appropriate care for unfamiliar diseases and to recognize emotional health problems, particularly when they are related to past experiences of violence."

Eventually, interventions for these children need to take into account the interfaces of institutional systems which these children and adolescents are living in. They are considered as refugees or foreigners by legislative systems, the psychological perspective – regarding them primarily as children [28]-, though, needs to be centred to address their primary mental health needs.

## Limitations

By definition, the current review is an overall analysis which involves, as a major limitation, the problem of bias in selection. First, the samples under study contain a large proportion of males. This is likely a product of the stresses involved in making long-distance journeys and the likelihood that girls would be at substantial risk for interpersonal and sexual violence if they undertook similar journeys. It is unclear if this factor alone would introduce bias because the URMs are the children and adolescents who arrive in the country of refuge, and therefore the gender imbalance may simply reflect the realities of this population. Second, this review may be limited by the quality of individual studies. Eventually, a considerable proportion of URMs in the mentioned studies are involved in support programmes whose major foci are interventions for these youth. This might produce considerable selection bias in that help-seeking youth are more likely to be sampled than those who might be avoidant. It is likely that the more psychiatrically morbid and the higher functioning youth might be selected out due to impairment and lack of need for services, respectively. Consequently, considerations about the profiles of psychopathology among these adolescents need to examine if the studies have focused on the most or least resilient URMs.

## Conclusion

URMs are a highly diverse group. The different ethnic backgrounds, their possible influence or upbringing, temperament and character along with the multiple motivations to leave the country of origin affect psychopathology parameters.

To this point, the literature has had a considerable emphasis on the assessment of PTSD symptoms, and reported higher levels of PTSD symptoms in comparison to the norm population and accompanied refugee minors. Despite studies such as the publication by Hodes et al. [13] which provide a comprehensive overview of past research and current issues in this field in addition to novel findings, the quality of the literature continues to be limited.

The following issues pose the most essential gaps of current literature. There exists a need to broaden the perspective by integrating models of coping with stress, personality profiles, long-term outcomes, resilience [40] and the examination of the full range of psychopathology in this vulnerable patient group. In addition, there is a need to examine the influence of legislative systems on the emotional and behavioural well-being of these children and adolescents. This consideration must address issues such as the absence of employment during the period of asylum seeking, the analysis of care systems (need for trained personnel, need for psychological support etc.) and the vulnerability of these youths for antisocial trajectories.

Additionally, there was a considerable lack of cultural sensitivity in the study designs. Thus, the existing literature only permits limited conclusions on this very hard to reach population. The development of culturally sensitive measures and standardized instruments for multi-cultural populations is of utmost importance and an indispensable component in adequate mental health care. Eventually, the selection of URMs in research needs to be carried out carefully in order to prevent selection bias, such as including only URMs who display resiliency.

In terms of interventions in the field of mental health issues among URMs, it is essential to apply multi-modal [41] and culturally sensitive methods. These children and adolescents belong to a diverse group but are collectively vulnerable due to their difficult legislative situation, their stressful past, and the sensitive developmental period of their lives during which these events are occurring.

## Competing interests

The authors declare that they have no competing interests.

## Authors' contributions

This review was designed and written by JH. The conception of the article and the acquisition of data was supervised by SV-K, KD, EG, MF, NK and HS. The analysis and interpretation of data was supervised by HS and NK. HS and NK were essentially involved in drafting the manuscript, revised it critically for important intellectual content and gave final approval of the version to be published.

## Supplementary Material

Additional file 1 **Numbers of arrivals of separated children and adolescents in Europe in 2006. Source: "Separated Children in Europe Programme" **[6]. The table provides information on numbers of arrivals of separated children and adolescents in Europe in 2006 [6]. Click here for file

Additional file 2 **Countries of origin of unaccompanied refugee minors, arriving in the United States of America between 1999 and 2005. Source: Department of Health and Human Services, Office of Refugee Resettlement, 2007 **[8]. The table provides information countries of origin of unaccompanied refugee minors, arriving in the United States of America between 1999 and 2005. Click here for file

Additional file 3 **Review of publications emphasizing URMs (1998–2008)**. The table summarizes publications emphasizing URMs (1998–2008). Click here for file

Additional file 4 **Non-migrants and migrant youth – Results on subscales of HSCL-37A, SDQ and RATS (Derluyn et al. **[16]). The table summarizes a study by Derluyn. Click here for file
