# Supplementary material for: Mental health issues in unaccompanied refugee minors
Source: Child Adolesc Psychiatry Ment Health. 2009 Apr 2;3:13. doi: 10.1186/1753-2000-3-13 (PMC2682790; doi:10.1186/1753-2000-3-13)
Supplement: Additional file 1 — Numbers of arrivals of separated children and adolescents in Europe in 2006. Source: "Separated Children in Europe Programme" [6]. The table provides information on numbers of arrivals of separated children and adolescents in Europe in 2006 [6]. [file 1753-2000-3-13-S1.doc]

**Table 1.** Numbers of arrivals of separated children and adolescents in Europe in 2006. Source: “Separated Children in Europe Programme” [6].

| ***Receiving country*** | ***Total*** | ***Main country of origin*** |
| --- | --- | --- |
| Austria | 488 (1) | Russian Fed., Afghanistan, Nigeria and Serbia |
| Belgium | 441 | Afghanistan, DR Congo, Guinea, Russia and Angola |
| Bulgaria | n | Afghanistan, Iraq, Iran,  Bangladesh and Nigeria |
| Croatia | 4 (a) | India and Sri Lanka |
| Denmark | 104 (a) | Iraq, Afghanistan, Iran and Nigeria |
| Estonia | 0 (a) |  |
| Finland | 108 | Iraq, Angola, Afghanistan, DR Congo, Somalia, Ethiopia and Iran |
| Germany | 186 (2) | Ethiopia, Vietnam, Afghanistan, Pakistan, Guinea, Iraq, Cambodia, Eritrea, Ivory Coast, Russian Fed. and Somalia |
| Ireland | n | n |
| Italy | 6551 (e) (3) | Romania, Morocco and Albania |
| Luxembourg | n | Algeria, Guinea, Somalia, Afghanistan, Russia, Burundi, Cameroon, Iran  and Zimbabwe |
| Malta | n | Somalia, Ethiopia, Sudan, Eritrea, Nigeria, Ghana and Burkina Faso |
| Netherlands | n | Somalia, Iraq, Afghanistan, Guinea and India |
| Norway | 349 (a) | Somalia, Iraq, Afghanistan, Sri Lanka, Ethiopia and Russia |
| Romania | 9 | Somalia and the Russian Federation |
| Slovakia | 138 (a) | India, Moldova, Pakistan, Bangladesh and China |
| Slovenia | 26 (e) | Albania, Serbia (Kosovo) and Turkey |
| Sweden | 820 | Iraq, Afghanistan and Somalia |
| Switzerland | 257 | Iraq, Ivory Coast, Afghanistan, Eritrea and Somalia |
| UK | 3,460 | Afghanistan, Iraq, Iran, Somalia and Eritrea |
| Total arrivals for 20 countries | 12,941 |  |

n = not available; a = actual; e = estimated.

(1) The official number is 74 cases lower. For those youth, the competent authority did not believe the age mentioned by the asylum seekers and added them to the group of adults.

(2) This number exclusively refers to minors up to the age of sixteen years. The Germany Residence Act determines legal competence in terms of asylum and residence law for minors between 16 and 18 years of age.

(3) SCEP assumes that this number is actually underestimated, since official statistics only include those youth who have been in touch with an Italian institution. Apart from that, SCEP mentions that Italy does not specify the age of the asylum applicant in official data reports.
