# Supplementary material for: Mental health issues in unaccompanied refugee minors
Source: Child Adolesc Psychiatry Ment Health. 2009 Apr 2;3:13. doi: 10.1186/1753-2000-3-13 (PMC2682790; doi:10.1186/1753-2000-3-13)
Supplement: Additional file 2 — Countries of origin of unaccompanied refugee minors, arriving in the United States of America between 1999 and 2005. Source: Department of Health and Human Services, Office of Refugee Resettlement, 2007 [8]. The table provides information countries of origin of unaccompanied refugee minors, arriving in the United States of America between 1999 and 2005. [file 1753-2000-3-13-S2.doc]

**Table 2.** Countries of origin of unaccompanied refugee minors, arriving in the United States of America between 1999 and 2005. Source: Department of Health and Human Services, Office of Refugee Resettlement, 2007 [8].

| ***Country*** | **Number of Minors** |
| --- | --- |
| ***Afghanistan*** | 30 |
| ***Africa, unspecified*** | 1 |
| ***Albania*** | 3 |
| ***Angola*** | 1 |
| ***Azerbaijan*** | 1 |
| ***Bosnia*** | 6 |
| ***Burundi*** | 4 |
| ***Cameroon*** | 1 |
| ***China*** | 20 |
| ***Congo*** | 12 |
| ***Cuba*** | 8 |
| ***El Salvador*** | 2 |
| ***Eritrea*** | 5 |
| ***Ethiopia*** | 8 |
| ***Guatemala*** | 11 |
| ***Guinea*** | 1 |
| ***Haiti*** | 56 |
| ***Honduras*** | 39 |
| ***India*** | 1 |
| ***Iran*** | 5 |
| ***Iraq*** | 1 |
| ***Kosovo*** | 4 |
| ***Liberia*** | 63 |
| ***Mexico*** | 10 |
| ***Nicaragua*** | 6 |
| ***Russia*** | 1 |
| ***Rwanda*** | 1 |
| ***Sierra Leone*** | 14 |
| ***Somalia*** | 27 |
| ***Sri Lanka*** | 1 |
| ***Sudan*** | 407 |
| ***Thailand*** | 2 |
| ***Vietnam*** | 26 |
| ***Yemen*** | 1 |
| ***Yugoslavia*** | 2 |
| ***Zimbabwe*** | 1 |
| ***Total*** | 782 |
