# Supplementary material for: Mental health issues in unaccompanied refugee minors
Source: Child Adolesc Psychiatry Ment Health. 2009 Apr 2;3:13. doi: 10.1186/1753-2000-3-13 (PMC2682790; doi:10.1186/1753-2000-3-13)
Supplement: Additional file 3 — Review of publications emphasizing URMs (1998–2008). The table summarizes publications emphasizing URMs (1998–2008). [file 1753-2000-3-13-S3.doc]

Table 3. Review of publications emphasizing URMs (1998-2008).

| Authors | Title | Year and journal of publication | Sample size | Location | Age group (yrs.) | Measures | Short summary |
| --- | --- | --- | --- | --- | --- | --- | --- |
| Bates L, Baird D, Johnson DJ, [Lee RE](http://www.ncbi.nlm.nih.gov/sites/entrez?Db=pubmed&Cmd=Search&Term="Lee RE"%5BAuthor%5D&itool=EntrezSystem2.PEntrez.Pubmed.Pubmed_ResultsPanel.Pubmed_DiscoveryPanel.Pubmed_RVAbstractPlus), [Luster T](http://www.ncbi.nlm.nih.gov/sites/entrez?Db=pubmed&Cmd=Search&Term="Luster T"%5BAuthor%5D&itool=EntrezSystem2.PEntrez.Pubmed.Pubmed_ResultsPanel.Pubmed_DiscoveryPanel.Pubmed_RVAbstractPlus), [Rehagen C](http://www.ncbi.nlm.nih.gov/sites/entrez?Db=pubmed&Cmd=Search&Term="Rehagen C"%5BAuthor%5D&itool=EntrezSystem2.PEntrez.Pubmed.Pubmed_ResultsPanel.Pubmed_DiscoveryPanel.Pubmed_RVAbstractPlus) | Sudanese refugee youth in foster care: the "lost boys" in America | Child Welfare 2005 | Five focus groups with 33 Sudanese minor youth | USA | < 18 | Child PTSD Symptom Scale, qualitative interviews | Need for increased funding to support more intensive educational services, more cultural training, foster parents, school personnel and flexibility to provide services in more culturally appropriate modalities when taking into account the challenges of adjusting to school and family life. |
| Bean T, Eurelings-Bontekoe L, Spinhoven P | Course and predictors of mental health of unaccompanied refugee minors in the Netherlands: one year follow-up | Soc Sci Med 2007 | 920 URMs, follow up: 582 URMs | Netherlands | 12-18 | HSCL-37A*, Stressful Life Events checklist, Reactions of Adolescents to Traumatic Stress, Child Behavioral Checklist Guardian Report, Teacher Report Form (TRF). | Chronic course of traumatic stress reactions. Elevated levels of stress reactions were in significant contrast to normative populations. Baseline psychopathology predicted psychological distress at follow-up. |
| Bean T, Derluyn I, Eurelings-Bontekoe L, Broekaert E, Spinhoven P | Comparing psychological distress, traumatic stress reactions, and experiences of unaccompanied refugee minors with experiences of adolescents accompanied by parents | J Nerv Ment Dis 2007 | Dutch URMs (n=920), Dutch normative population (n=1059), Belgian immigrant and refugee adolescents (n=1294) | Netherlands | 13-18 | HSCL-37A*, Stressful Life Events (SLE), Reactions of Adolescents to Traumatic Stress (RATS) | Gender played a modest role in internalizing emotional problems and externalizing behaviour in the two comparison groups but not among URMs. The older the adolescent, the more emotional problems and the more experienced stressful life events were reported. URMs showed a higher number of average stressful life events. |
| [Bean T](http://www.ncbi.nlm.nih.gov.laneproxy.stanford.edu/sites/entrez?Db=pubmed&Cmd=Search&Term="Bean T"%5BAuthor%5D&itool=EntrezSystem2.PEntrez.Pubmed.Pubmed_ResultsPanel.Pubmed_DiscoveryPanel.Pubmed_RVAbstractPlus), [Derluyn I](http://www.ncbi.nlm.nih.gov.laneproxy.stanford.edu/sites/entrez?Db=pubmed&Cmd=Search&Term="Derluyn I"%5BAuthor%5D&itool=EntrezSystem2.PEntrez.Pubmed.Pubmed_ResultsPanel.Pubmed_DiscoveryPanel.Pubmed_RVAbstractPlus), [Eurelings-Bontekoe E](http://www.ncbi.nlm.nih.gov.laneproxy.stanford.edu/sites/entrez?Db=pubmed&Cmd=Search&Term="Eurelings-Bontekoe E"%5BAuthor%5D&itool=EntrezSystem2.PEntrez.Pubmed.Pubmed_ResultsPanel.Pubmed_DiscoveryPanel.Pubmed_RVAbstractPlus), [Broekaert E](http://www.ncbi.nlm.nih.gov.laneproxy.stanford.edu/sites/entrez?Db=pubmed&Cmd=Search&Term="Broekaert E"%5BAuthor%5D&itool=EntrezSystem2.PEntrez.Pubmed.Pubmed_ResultsPanel.Pubmed_DiscoveryPanel.Pubmed_RVAbstractPlus), [Spinhoven P](http://www.ncbi.nlm.nih.gov.laneproxy.stanford.edu/sites/entrez?Db=pubmed&Cmd=Search&Term="Spinhoven P"%5BAuthor%5D&itool=EntrezSystem2.PEntrez.Pubmed.Pubmed_ResultsPanel.Pubmed_DiscoveryPanel.Pubmed_RVAbstractPlus) | Validation of the multiple language versions of the Hopkins Symptom Checklist-37 for refugee adolescents | Adolescence 2007 | Dutch URMs (n=920), Belgian normative population (n= 617), Dutch normative population (n=1059), Belgian immigrant and refugee adolescents (n=1294) | Netherlands | 13-18 | Validation study | Modifications were made to the instrument in order to assess internalizing and externalizing symptoms associated with reactions to trauma, and to make it accessible to culturally diverse, adolescent populations. The questionnaires were translated and presented bilingually. The confirmative two factor analyses, by language version, re-established the two factor structure of internalizing and externalizing symptoms. The total scale as well as the subscales showed good internal consistency and acceptable test-rest reliability. Cronbach’s alpha for the total score was r= .9, it ranged from r= .95 to r= .84 for the language versions. Apart from that, the construct, content and criterion validity were rated as good. The authors stated that the HSCL-37A was a reliable and valid instrument among culturally diverse refugee adolescents to assess emotional distress and maladaptive behaviour. |
| [Bean T](http://www.ncbi.nlm.nih.gov.laneproxy.stanford.edu/sites/entrez?Db=pubmed&Cmd=Search&Term="Bean T"%5BAuthor%5D&itool=EntrezSystem2.PEntrez.Pubmed.Pubmed_ResultsPanel.Pubmed_DiscoveryPanel.Pubmed_RVAbstractPlus), [Derluyn I](http://www.ncbi.nlm.nih.gov.laneproxy.stanford.edu/sites/entrez?Db=pubmed&Cmd=Search&Term="Derluyn I"%5BAuthor%5D&itool=EntrezSystem2.PEntrez.Pubmed.Pubmed_ResultsPanel.Pubmed_DiscoveryPanel.Pubmed_RVAbstractPlus), [Eurelings-Bontekoe E](http://www.ncbi.nlm.nih.gov.laneproxy.stanford.edu/sites/entrez?Db=pubmed&Cmd=Search&Term="Eurelings-Bontekoe E"%5BAuthor%5D&itool=EntrezSystem2.PEntrez.Pubmed.Pubmed_ResultsPanel.Pubmed_DiscoveryPanel.Pubmed_RVAbstractPlus), [Broekaert E](http://www.ncbi.nlm.nih.gov.laneproxy.stanford.edu/sites/entrez?Db=pubmed&Cmd=Search&Term="Broekaert E"%5BAuthor%5D&itool=EntrezSystem2.PEntrez.Pubmed.Pubmed_ResultsPanel.Pubmed_DiscoveryPanel.Pubmed_RVAbstractPlus), [Spinhoven P](http://www.ncbi.nlm.nih.gov.laneproxy.stanford.edu/sites/entrez?Db=pubmed&Cmd=Search&Term="Spinhoven P"%5BAuthor%5D&itool=EntrezSystem2.PEntrez.Pubmed.Pubmed_ResultsPanel.Pubmed_DiscoveryPanel.Pubmed_RVAbstractPlus) | Validation of the multiple language versions of the Reactions of Adolescents to Traumatic Stress questionnaire | J Trauma Stress 2006 | Dutch URMs (n=920), Belgian normative population (n= 617), Dutch normative population (n=1059), Belgian immigrant and refugee adolescents (n=939) | Netherlands | 13-18 | Validation study | The authors carried out similar modifications as in the previously described study examining the HSCL-37A. The confirmatory factor analyses, by language version, gave support to a three-factor structure of intrusion, avoidance/numbing, and hyperarousal. The total and subscales of the RATS showed good internal consistency and validity. Cronbach’s alpha for the total score was r= .91, it ranged from r= .81 to r= .93 for the language versions. The RATS was described as a reliable and valid instrument for assessing posttraumatic stress reactions of culturally diverse adolescents. |
| [Bean T](http://www.ncbi.nlm.nih.gov.laneproxy.stanford.edu/sites/entrez?Db=pubmed&Cmd=Search&Term="Bean T"%5BAuthor%5D&itool=EntrezSystem2.PEntrez.Pubmed.Pubmed_ResultsPanel.Pubmed_DiscoveryPanel.Pubmed_RVAbstractPlus), [Eurelings-Bontekoe E](http://www.ncbi.nlm.nih.gov.laneproxy.stanford.edu/sites/entrez?Db=pubmed&Cmd=Search&Term="Eurelings-Bontekoe E"%5BAuthor%5D&itool=EntrezSystem2.PEntrez.Pubmed.Pubmed_ResultsPanel.Pubmed_DiscoveryPanel.Pubmed_RVAbstractPlus), [Mooijaart A](http://www.ncbi.nlm.nih.gov.laneproxy.stanford.edu/sites/entrez?Db=pubmed&Cmd=Search&Term="Mooijaart A"%5BAuthor%5D&itool=EntrezSystem2.PEntrez.Pubmed.Pubmed_ResultsPanel.Pubmed_DiscoveryPanel.Pubmed_RVAbstractPlus), [Spinhoven P](http://www.ncbi.nlm.nih.gov.laneproxy.stanford.edu/sites/entrez?Db=pubmed&Cmd=Search&Term="Spinhoven P"%5BAuthor%5D&itool=EntrezSystem2.PEntrez.Pubmed.Pubmed_ResultsPanel.Pubmed_DiscoveryPanel.Pubmed_RVAbstractPlus) | Factors associated with mental health service need and utilization among unaccompanied refugee adolescents | Adm Policy Ment Health 2006 | URMs (n = 920), legal guardians (n = 557), teachers (n = 496), Dutch adolescents (n = 1059) | Netherlands | URMs: Mean age = 15.48, SD=1.52;  Dutch adolescents:  Mean age=15.72, SD=1.54 | HSCL-37A*, RATS SLE, CBCL, TRF, mental health questionnaires for guardians/teachers | The referral of URM to mental health care services seems to be influenced by the need and emotional distress as noticed by guardians. Consequently, 48.7% of the URM total sample reported that their need for mental health care was not sufficiently met. |
| Derluyn I, Broekaert E, Schuyten G | Emotional and behavioural problems in migrant adolescents in Belgium | Eur Child Adolesc Psychiatry 2008 | Migrant (N=1249) and Belgian (N=602) adolescents | Belgium | 11-18 | HSCL-37A*, SLE, SDQ (Strengths and Difficulties Questionnaire) and RATS | URMs – as part of the migrant adolescents in the study population - reported more emotional problems and more symptoms of anxiety, depression and PTSD, but less conduct problems than accompanied migrant adolescents. According to the outcomes of the study, the time a migrant adolescent was living in Belgium did not influence the prevalence of emotional and behavioural problems. |
| Derluyn I, Broekaert E | Unaccompanied refugee children and adolescents: The glaring contrast between a legal and a psychological perspective | Int J Law Psychiatry 2008 | Qualitative approach | Belgium |  | Health service study  with interventional approaches | URMs should be primarily considered as children and not as refugees or foreigners as emphasized by legislative frameworks. |
| [Derluyn I](http://www.ncbi.nlm.nih.gov.laneproxy.stanford.edu/sites/entrez?Db=pubmed&Cmd=Search&Term="Derluyn I"%5BAuthor%5D&itool=EntrezSystem2.PEntrez.Pubmed.Pubmed_ResultsPanel.Pubmed_DiscoveryPanel.Pubmed_RVAbstractPlus), [Broekaert E](http://www.ncbi.nlm.nih.gov.laneproxy.stanford.edu/sites/entrez?Db=pubmed&Cmd=Search&Term="Broekaert E"%5BAuthor%5D&itool=EntrezSystem2.PEntrez.Pubmed.Pubmed_ResultsPanel.Pubmed_DiscoveryPanel.Pubmed_RVAbstractPlus). | Different perspectives on emotional and behavioural problems in unaccompanied refugee children and adolescents | [Ethn Health](javascript:AL_get(this, 'jour', 'Ethn Health.');) 2007 | 166 URMs | Belgium | 12-18 | HSCL-37A*, SDQ-self, RATS SLE, CBCL (Child Behavior Checklist) and SDQ-parent | Results revealed that between 37-47% of 166 URMs suffered from severe or very severe symptoms of anxiety, depression and posttraumatic stress. Girls and those who had experienced greater numbers of traumatic events were at higher risk for the development of emotional problems. Social workers reported a high prevalence of internalising problems and important externalising problems in the study population. |
| [Geltman PL](http://www.ncbi.nlm.nih.gov/sites/entrez?Db=pubmed&Cmd=Search&Term="Geltman PL"%5BAuthor%5D&itool=EntrezSystem2.PEntrez.Pubmed.Pubmed_ResultsPanel.Pubmed_DiscoveryPanel.Pubmed_RVAbstractPlus), [Grant-Knight W](http://www.ncbi.nlm.nih.gov/sites/entrez?Db=pubmed&Cmd=Search&Term="Grant-Knight W"%5BAuthor%5D&itool=EntrezSystem2.PEntrez.Pubmed.Pubmed_ResultsPanel.Pubmed_DiscoveryPanel.Pubmed_RVAbstractPlus), [Ellis H](http://www.ncbi.nlm.nih.gov/sites/entrez?Db=pubmed&Cmd=Search&Term="Ellis H"%5BAuthor%5D&itool=EntrezSystem2.PEntrez.Pubmed.Pubmed_ResultsPanel.Pubmed_DiscoveryPanel.Pubmed_RVAbstractPlus), [Landgraf JM](http://www.ncbi.nlm.nih.gov/sites/entrez?Db=pubmed&Cmd=Search&Term="Landgraf JM"%5BAuthor%5D&itool=EntrezSystem2.PEntrez.Pubmed.Pubmed_ResultsPanel.Pubmed_DiscoveryPanel.Pubmed_RVAbstractPlus) | The "lost boys" of Sudan: use of health services and functional health outcomes of unaccompanied refugee minors resettled in the U.S. | [J Immigr Minor Health](javascript:AL_get(this, 'jour', 'J Immigr Minor Health.');) 2008 | 304 male Sudanese URMs | USA | Mean age = 17.6 | Child Health Questionnaire (CHQ), questions regarding care for symptoms associated with behavioural disorders, Harvard Trauma Questionnaire (HTQ) | The adolescents described high rates of counseling (45%). The majority of the assessed group (76%) described that they sought medical care for problems associated with behavioural and emotional issues; this was more common among the adolescents who suffered from PTSD. Those URMs with worse functional health weren’t more likely to have received mental health counseling. At the same time, though, they were more likely to seek care from any health professional, a fact that was also true for those adolescents with PTSD. |
| Geltman PL, Grant-Knight W, Ellis H, Landgraf | The "Lost Boys of Sudan". Functional and behavioral health of unaccompanied refugee minors resettled in the United States | Arch Pediatr Adolesc Med 2005 | 304 male Sudanese URMs | USA | Mean age = 17.6 | Open interviews, Child Health Questionnaire, Harvard Trauma Questionnaire, Ways of Coping Instrument, YSR (Youth Self-Report) | Twenty per cent of the adolescents suffered from PTSD; these adolescents were more likely to have significantly worse functional scores in all Child Health Questionnaire subscales. In addition, social isolation and personal injury were associated with PTSD. |
| Goodman JH | Coping with trauma and hardship among unaccompanied refugee youths from Sudan. | Qual Health Res 2004 | 14 male URMs | USA | 16-18 | Case-centred, comparative, narrative approach to analyze transcripts | Qualitative outcomes in terms of coping strategies. |
| [Hodes M](http://www.ncbi.nlm.nih.gov/sites/entrez?Db=pubmed&Cmd=Search&Term="Hodes M"%5BAuthor%5D&itool=EntrezSystem2.PEntrez.Pubmed.Pubmed_ResultsPanel.Pubmed_DiscoveryPanel.Pubmed_RVAbstractPlus), [Jagdev D](http://www.ncbi.nlm.nih.gov/sites/entrez?Db=pubmed&Cmd=Search&Term="Jagdev D"%5BAuthor%5D&itool=EntrezSystem2.PEntrez.Pubmed.Pubmed_ResultsPanel.Pubmed_DiscoveryPanel.Pubmed_RVAbstractPlus), [Chandra N](http://www.ncbi.nlm.nih.gov/sites/entrez?Db=pubmed&Cmd=Search&Term="Chandra N"%5BAuthor%5D&itool=EntrezSystem2.PEntrez.Pubmed.Pubmed_ResultsPanel.Pubmed_DiscoveryPanel.Pubmed_RVAbstractPlus), Cunniff A | Risk and resilience for psychological distress amongst unaccompanied asylum seeking adolescents | [J Child Psychol Psychiatry](javascript:AL_get(this, 'jour', 'J Child Psychol Psychiatry.');) 2008 | 78 URMs, 35 accompanied RMs | UK | 13-18 | Birleson Depression Self-Rating Scale, Impact of Event Scale (IES), Harvard Trauma Questionnaire | URMs had been affected by high levels of war trauma and losses and had elevated posttraumatic stress symptoms. Low-support living circumstances, trauma events, increasing age and gender predicted posttraumatic symptoms only among unaccompanied refugee youths. |
| Loughry M, Flouri E | The behavioral and emotional problems of former unaccompanied refugee children 3-4 years after their return to Vietnam. | Child Abuse Negl 2001 | 455, 233 of them were URMs – compared to non-refugees who had never left Vietnam | Vietnam | 10-22 | Youth Self-Report, Cowen Perceived Self-Efficacy scale, Social Support Scale, Exposure to Traumatic Events Scale | The two groups differed on the externalizing, but not on the internalizing or total problem scores. Those who described their current standard of living as ‘very difficult’ reported the highest internalizing scores. |
| Lynch MA | Providing health care for refugee children and unaccompanied minors | [Med Confl Surviv](javascript:AL_get(this, 'jour', 'Med Confl Surviv.');) 2001 | Qualitative approach |  |  | Health service study | The need for recognition and treatment of emotional problems beyond the mere access to basic health care is outlined. |
| Reijneveld SA, De Boer JB, Bean T, Korfker DG | Unaccompanied adolescents seeking asylum: poorer mental health under a restrictive reception | J Nerv Ment Dis 2005 | 122 URMs | Netherlands | 15-18 | Hopkins Symptom Checklist-25, RATS | More emotional problems (Hopkins Symptom Checklist) were found among adolescents in the restricted reception centre. |
| Rousseau C, Said TM, Gagne MJ, Bibeau G | Resilience in unaccompanied minors from the north of Somalia | [Psychoanal Rev](javascript:AL_get(this, 'jour', 'Psychoanal Rev.');) 1998 | 10 URMs | Canada | 13-18 | Qualitative interviews | Concepts of resilience are discussed in the framework of two normative systems including the traditional upbringing in Somali culture and the forced exile experience in North America. |
| [Sourander A](http://www.ncbi.nlm.nih.gov/sites/entrez?Db=pubmed&Cmd=Search&Term="Sourander A"%5BAuthor%5D&itool=EntrezSystem2.PEntrez.Pubmed.Pubmed_ResultsPanel.Pubmed_DiscoveryPanel.Pubmed_RVAbstractPlus) | Behavior problems and traumatic events of unaccompanied refugee minors | [Child Abuse Negl](javascript:AL_get(this, 'jour', 'Child Abuse Negl.');), 1998 | 46 URMs | Finland | Mean age = 14.1 | Clinical information, CBCL | Approximately half of the adolescents had results within clinical or borderline range (CBCL). Qualitative outcomes. |
| Spinhoven P, Bean T, Eurelings-Bontekoe L | Inconsistencies in the self-report of traumatic experiences by unaccompanied refugee minors | J Trauma Stress 2006 | 920 URMs, follow up: 582 URMs | Netherlands | 12-18 | HSCL-37A*, Stressful Life Events checklist, Reactions of Adolescents to Traumatic Stress | Younger participants and those with lower levels of internalizing behaviour and posttraumatic stress at follow-up were at higher risk of memory inconsistencies. |
| Thomas S, Thomas S, Nafees B, Bhugra D | 'I was running away from death'- the pre-flight experiences of unaccompanied asylum seeking children in the UK. | [Child Care Health Dev](javascript:AL_get(this, 'jour', 'Child Care Health Dev.');) 2004 | 120 URMs | UK | 11-17 | Analysis of legal statements, case notes and interviews | Nearly half of all URMs had experienced separation from or loss of parents and/or family members (47%), and a further 41% had personally experienced or witnessed violence. Sexual violence (such as rape) was reported by 24% of African girls. |
| Wallin AM, Ahlstroem GI | Unaccompanied young adult refugees in Sweden, experiences of their life situation and well-being: a qualitative follow-up study | Ethn Health 2005 | 34 URMs | Sweden | 16-26, follow up after ten years | Qualitative interview | The majority of the participants had worked through the problems that typically affect refugees and had started to adapt to their new country, while a few still felt alone and set apart from the community. |
| Wiese EB, Burhorst I | The mental health of asylum-seeking and refugee children and adolescents attending a clinic in the Netherlands | Transcult Psychiatry 2007 | 70 accompanied RMs, 59 URMs | Netherlands | 0-18+ | Intake report, structured questionnaire | Significant difference in traumatic events. URMs were more likely to have been victim to four or more traumatic events. They had a significantly higher prevalence of depressive disorder, borderline personality disorder, and psychosis. |

* Hopkins Symptom Checklist-37 for Adolescents
