# Supplementary material for: Mental health issues in unaccompanied refugee minors
Source: Child Adolesc Psychiatry Ment Health. 2009 Apr 2;3:13. doi: 10.1186/1753-2000-3-13 (PMC2682790; doi:10.1186/1753-2000-3-13)
Supplement: Additional file 4 — Non-migrants and migrant youth – Results on subscales of HSCL-37A, SDQ and RATS (Derluyn et al. [16]). The table summarizes a study by Derluyn. [file 1753-2000-3-13-S4.doc]

| **Measures** | **Subscales** | **Non-migrants** | **Migrants** | **p** |
| --- | --- | --- | --- | --- |
| HSCL-37A | Anxiety | 1.77 | 1.66 | < 0.05 |
|  | Externalizing | 1.56 | 1.28 | < 0.001 |
| SDQ | Hyperactivity | 4.75 | 3.04 | < 0.04 |
|  | Peer problems | 2.09 | 2.86 | < 0.001 |
| RATS | Avoidance | 14.81 | 16.44 | < 0.04 |

Table 4. Non-migrants and migrant youth – Results on subscales of HSCL-37A, SDQ and RATS (Derluyn et al. [16]).
